# High school football player experiences with multiple injuries: a qualitative biopsychosocial model application

**DOI:** 10.3389/fspor.2025.1583467

**Published:** 2025-06-19

**Authors:** Natalie Golub, Jesse A. Steinfeldt

**Affiliations:** Department of Applied Psychology in Education & Research Methodology, Indiana University, Bloomington, IN, United States

**Keywords:** injury, biopsychosocial, football, qualitative, high school

## Abstract

**Introduction:**

High school athletes in the United States sustain approximately 1.3 million sport-related injuries annually, with nearly half occurring in football. These injuries can significantly impact athletes' psychological and behavioral well-being, influenced by factors such as athletic identity, self-efficacy, prosocial behavior, and prior injury history. While the Biopsychosocial Model of Sport Injury Rehabilitation offers a comprehensive framework for understanding injury recovery, limited research has examined how athletes respond to multiple injuries over time.

**Methods:**

This qualitative study applied the Biopsychosocial Model to explore the lived experiences of eight male high school football players who sustained multiple injuries during a single season. Each participant missed at least one week of play and/or one game per injury. Semi-structured interviews were conducted to investigate emotional responses, perceived social support, and stress management. Thematic analysis was used to analyze the data, following an inductive approach that allowed themes to emerge organically from participants' narratives.

**Results:**

Participants shared detailed accounts of their injuries, recovery processes, and the broader impacts on their lives. Thematic analysis revealed four overarching themes: (a) emotional response, (b) sources of support, (c) stress effects, and (d) coping strategies. Athletes described a wide range of emotional and behavioral responses, including frustration, anxiety, and determination. Support systems—such as family, coaches, and teammates—played a critical role in their recovery. Stress related to performance, identity, and future prospects was common, and athletes employed various coping mechanisms, including mental reframing, goal setting, and seeking social support. These responses were shaped by individual injury histories and personal resilience.

**Discussion:**

The findings highlight the complex and varied ways high school football players experience and manage multiple injuries. Emotional reactions, support networks, and coping strategies all play a role in shaping recovery outcomes. Understanding these lived experiences can inform more holistic and personalized approaches to injury rehabilitation. Interventions that address emotional well-being, enhance social support, and promote effective coping strategies may improve recovery and reduce the risk of future injuries.

## Introduction

High school-aged athletes in the U.S. are estimated to sustain more than 1.3 million sports-related injuries each year (1). Injuries are a part of almost any athlete's experience due to the physical demands required to compete at high levels. An athletic injury can be defined as a pathological process that interrupts training or competition and may lead the athlete to seek medical attention (2). How an athlete responds to injury differs greatly depending on factors such as athletic identity, self-efficacy, prosocial behaviors, previous injury history, and other pre-injury characteristics (3–5). Regardless of individual differences in response, sport injuries cause significant challenges for athletes and have the potential to affect long-term health, wellness, and performance.

Athletic injuries induce a range of biological, psychological, and social responses and consequences. The Biopsychosocial Model of Sport Injury Rehabilitation (6) offers a comprehensive framework to understand how athletes respond to injuries and how these responses impact rehabilitation outcomes. While various aspects of this model have been extensively studied, there remains a notable gap in understanding how an athlete's injury history influences other factors and the overall rehabilitation process. A particularly underexplored area is the impact of multiple injuries on athletes. Our study aims to empirically examine the lived experiences of high school football players who have sustained multiple injuries, utilizing a Biopsychosocial perspective.

### Injury overview: definitions and prevalence

Varying definitions of athletic injury exist due to variables such as time out of play, circumstances, location, and severity. The National Athletic Treatment, Injury and Outcomes Network Surveillance Program (NATION-SP) define a reportable injury as “an injury that (1) occurred as a result of participation in an organized HS-sanctioned athletic event for a school-sponsored sport and (2) required attention from an AT or physician, regardless of time loss” (7, p. 530). The current study defines athletic injury as any injury (regardless of severity, location, or circumstances) that results in missing one week of sport participation and/or one contest. Adding a stringent time out-of-play allows for clarity of instances of injury across a season for assessing multiple injury experiences.

However, defining *multiple injuries* proves challenging, given the diverse criteria involving severity, location, and injury mechanisms, primarily rooted in medical paradigms, which may not consistently match the dynamics of sports contexts (8, 9). Terms such as “repeat”, “recurrent”, and “multiple” are frequently employed interchangeably to denote instances of injury recurrence, with “recurrent/repeat” generally describing the repetition of a single injury, while “multiple injuries” signify the occurrence of several distinct and unrelated injuries. In this study, *multiple injuries* are defined as more than one occurrence of injury at different times resulting in a week of missed participation and/or a missed contest, including repeated or recurrent injuries. Despite their importance, multiple injuries are significantly underreported in injury prevalence literature. A systematic review of injury reporting in team ball sports found that half of the 71 publications reviewed did not specify multiple individual injuries, and those that did primarily focused on reporting recurrent injuries (10). This underreporting and lack of accurate representation in both research and practice limits our understanding of how athletes respond to and recover from new or subsequent injuries.

In the U.S., boys' football has the highest rate of sports-related injuries with 3.96 injuries per 1,000 athlete exposures (11). Welton et al. (12) observed that boys' football had the highest rates of both new (36.82) and recurrent (3.81) injuries per 10,000 athlete exposures from 2005 to 2016. Over the academic years 2005–2006 to 2015–2016, 10.5% of all high school sports injuries were recurrent, leading to increased time away from sport, heightened risk of discontinuation, and greater likelihood of surgical intervention compared to new injuries. This examination of athletic injury prevalence highlights significant gaps in the literature, particularly the lack of information on multiple injuries, with existing assessments limited to recurrent injuries, thus overlooking other instances of multiple injuries.

### Biopsychosocial model

**Figure 1:**
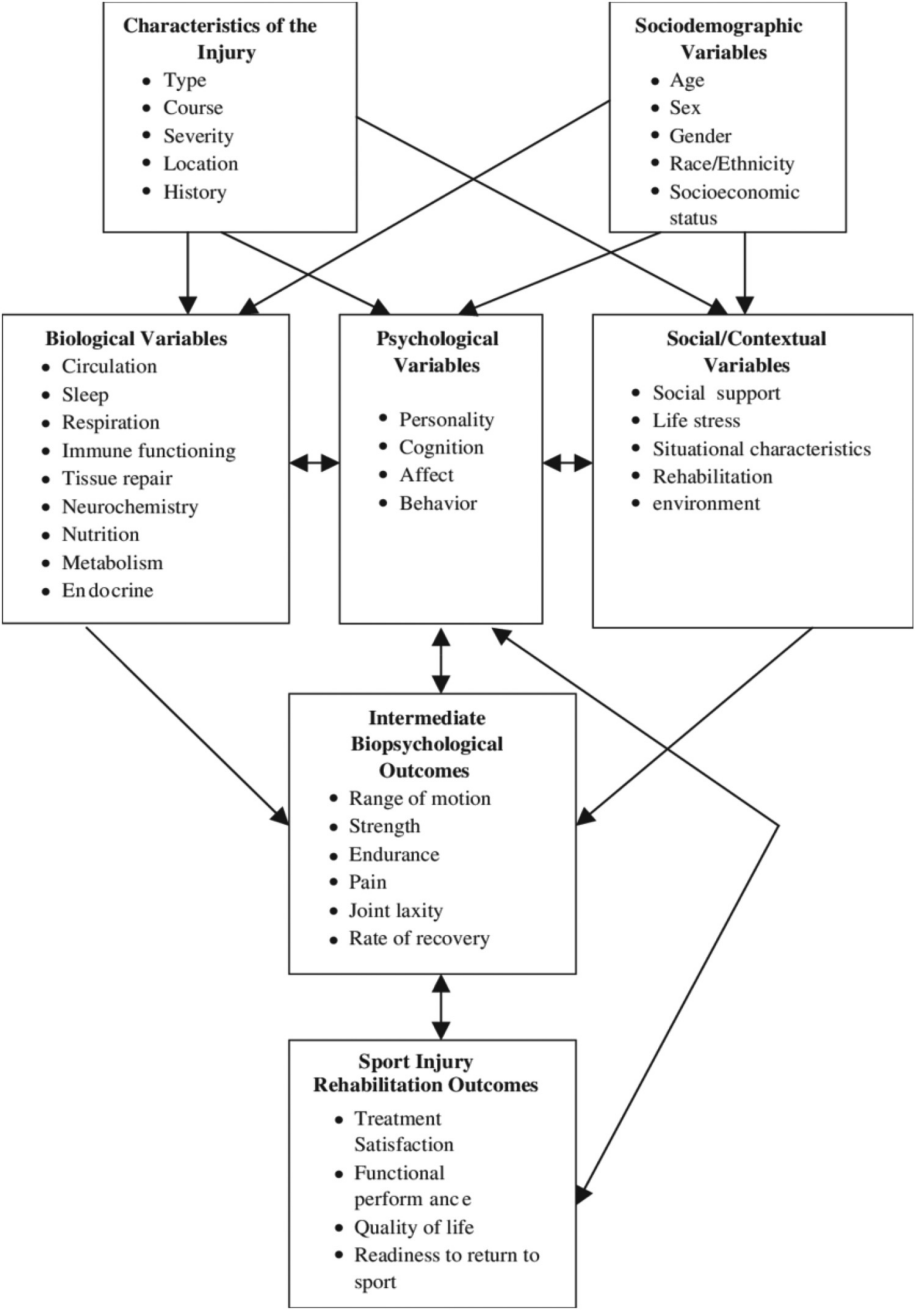
Biopsychosocial model of sport-injury rehabilitation (6).

Several models have been developed to understand factors influencing injury response (13). Initially, injuries were conceptualized through the Biomedical Model which theorizes that physical and mental illnesses are caused by physiological deviations from the norm (14). Expanding from this, the Biopsychosocial model advocates for a more holistic perspective, incorporating biological, psychological, and social factors to better understand injury and illness (15). Brewer et al. (6) extended this model specifically for sports injury rehabilitation, resulting in the creation of the Biopsychosocial Model of Sport-Injury Rehabilitation (see Figure 1). This integrated model comprises seven dimensions: injury characteristics, sociodemographic factors, biological factors, psychological factors, social and contextual factors, intermediate biopsychological outcomes, and sports injury rehabilitation outcomes. The model posits that the sport injury rehabilitation process begins with injury occurrence, encompassing factors like location, type, cause, severity, and injury history, all influencing biological, psychological, and social dimensions (16). Socio-demographic factors such as age, gender, ethnicity, and socioeconomic status also impact these dimensions. These factors collectively influence intermediate outcomes like muscle strength, joint flexibility, pain perception, and recovery duration, which in turn affect rehabilitation outcomes like functional performance, quality of life post-injury, treatment satisfaction, and return-to-sport readiness (17). Psychological factors play a central role, interacting reciprocally with biological and socio-contextual factors, and with intermediate and final outcomes, with all factors exhibiting bidirectional relationships. In applying the Biopsychosocial Model of Sport-Injury Rehabilitation (6) this study will assess biological, psychological, and social responses to multiple injuries.

#### Biological response

Injury triggers heightened arousal within the body, activating a stress response that induces neurological, hormonal, and metabolic changes affecting all bodily systems (18). Repeated exposure to high stress levels, such as with multiple injuries, can lead to chronic stress, limiting the body's ability to initiate the relaxation response. Chronic stress disrupts cortisol levels due to repeated cortisol surges, leading to dysregulation (19), which impairs an athlete's ability to engage in the arousal-relaxation cycle crucial for training and recovery, potentially hindering full recovery. Multiple injuries may exacerbate these detrimental effects on an athlete's injury response, recovery, and return to sport.

#### Psychological response

In addition to the physiological disruption of sports injuries, psychological effects include disruptions to cognition, emotion, and behavior (20). Many athletes interpret sports injuries as major negative life events (21–24), with experts viewing sudden and prolonged sports injury as contributors to poor mental health outcomes (25). Injuries can evoke emotional reactions such as depression, anger, fear, tension, disgust, anxiety, and panic (26–28). Furthermore, athletes with recent injuries exhibit greater negative cognitive content regarding reinjury expectations, show reduced confidence in injury prevention and increased perceived risk and worry about further injury compared to those without recent injuries (29).

Following injury, individuals often adopt altered behaviors as a means of coping with physical and psychological distress (20). Adaptive strategies for recovery include investing in rehabilitation, seeking information, exploring alternative treatments, building physical strength, and leveraging social support (30–32). Conversely, avoidant coping strategies, such as distracting and isolating oneself may be employed to regulate post-injury emotions (33). Maladaptive coping may involve risky behaviors such as suicide attempts, disordered eating, and substance use (23). The nature of the injury can influence coping behaviors, potentially affecting rehabilitation adherence and overall well-being.

#### Social response

Social support, broadly defined as social interactions aimed at inducing positive outcomes (34), is recognized as one of the most adaptive factors in injury recovery (35). Research suggests that social support serves as a significant coping mechanism (36), correlated with reduced psychological distress (37) and improved adherence to rehabilitation programs (38). Social support is multidimensional, encompassing structural (relationships), functional (exchanges), and perceptual (appraisal) components (39, 40). Structural aspects include important relationships within injured athletes' support network while functional aspects are the actions and functions served by these social relationships (41). Perception of support can be perceived support; an individual's subjective assessment of the availability of support (42, 43), or received support; the exchange of specific helping actions (44). These aspects, influenced by factors including sociocultural context and relationship characteristics, play a vital role in facilitating positive health outcomes such as successful injury rehabilitation and return to sport (34).

In analyzing various models and theories on injury response, it's clear that a comprehensive approach is crucial to understand the challenges athletes confront while injured. The injury modeling evolution has led to a growing recognition of the interplay among biological, psychological, and social factors in shaping injury outcomes. The Biopsychosocial Model (6) provides a structured framework for the current study to explore the distinct experiences of multiply injured athletes.

## Current study

This study explores the unique experiences of athletes with multiple injuries, aiming to fill a current gap in the literature. To date, there has been only one unpublished thesis specifically investigating this topic, conducted by Secrest (65), which focused on the psychological experiences of female collegiate athletes with multiple injuries. Results revealed emotions like shock, anger, frustration, and distrust in their bodies, highlighting the need for further research to develop effective support strategies for this group of athletes. The current study will expand on these findings, assessing emotional response, perceived social support, and stress perception and management through qualitative interviews with high school football players. This population is crucial to examine due to the high prevalence and severity of injuries in football, making athletes at increased risk for multiple injuries throughout their careers (1). This investigation involves post-injury semi-structured interviews exploring emotional responses, perceived social support, and stress perception and management constructs. This qualitative approach will allow us to explore: (a) how do male high school football players experience emotions related to multiple injuries? (b) What are male high school football players' perceptions of social support throughout their injuries? (c) How do high school football players experience and cope with stress following multiple injuries?

## Methods

Participants were recruited from a large longitudinal subconcussive impact study among five different high schools. Eight male high school football players who had sustained multiple injuries during a season participated in the present study. Ages ranged from 14 to 18 years (mean = 15.6, SD = 1.6) and they were freshmen (*n* = 2), sophomores (*n* = 3), juniors (*n* = 2), and a senior (*n* = 1)—six participants self-identified as White, one as Black, and one as mixed race. There was a total of 17 self-reported injuries sustained among participants including wrist injury, broken arm, concussion, broken thumb, dislocated shoulder, back injury, broken collar bone, pulled groin, and torn labrum in the shoulder. Pseudonyms were given to each participant and are described in Table 1 including individual injury characteristics.

**Table 1:** Participant characteristics.

Participant	Age	Race	Position	Injuries sustained- injury event	Severity (total time loss)
Danny	14	White	Lineman	Wrist bone bruise- tackling Broken arm- tackling	9 weeks (estimate)
Andrew	15	Black	Running back/quarterback	Concussion- during game Broken thumb- during game	5 weeks
John	15	White	Lineman	Two shoulder dislocations, same side- during games	4 weeks
Kyle	15	White	Tight end	Concussion- during practice Concussion- during game	4 weeks
Mike	14	White	Defensive end	Back strain- fall outside of football Broken collar bone- during game	9 ½ weeks
Zach	16	White	Lineman	Pulled groin- during practice Spinal trauma resulting in stroke- multiple in-game hits	4 weeks
Hunter	18	White	Corner	Concussion- during game Broken wrist- lifting weights Shoulder torn labrum- tackling	7 weeks
Tyler	18	Black and Asian	Corner	Two shoulder dislocations, same side- during games	5 months (estimate)

### Measures

#### Demographics

Self-report demographic questionnaires were administered in pre-season data collection. The information included in the present study is age, grade, and racial self-identification. Football-related data were collected including the number of years playing football and position. During the interviews, data on injury history were collected, including the number of injuries, type and location, circumstances, duration of time out of play, and rehabilitation details.

#### Interview protocol

Participants engaged in a semi-structured interview focusing on their injury experiences. The interview consisted of 11 open-ended questions with categories of essential questions, emotional response, social implications, stress, and other. The development of the interview protocol was guided by an extensive review of injury-related research and relevant qualitative studies. Additionally, consultations with clinicians, coaches, athletic trainers, and experts in the field played a crucial role in shaping the protocol. The interview structure and questions were developed based on existing literature in qualitative injury research and thematic analysis. Each of the constructs of emotional response, social implications, and stress questions were developed to align with theory and research in their respective areas. Interviews were conducted and recorded via Zoom and then transcribed.

The interviews were conducted by a female doctoral student in counseling and sport psychology. As a former athlete with training and practice in sport psychology, the interviewer brought a perspective shaped by both personal experience in competitive athletics and a professional commitment to athlete mental health and well-being. This background likely fostered deeper empathy for participants' experiences and an informed understanding of the cultural norms and pressures within sport contexts. At the same time, differences in age and gender between the interviewer and participants may have influenced the dynamics of the interviews, potentially affecting participants' openness or shaping the interpretation of their responses. The interviewer has both clinical and academic experience working with adolescent athletes and has received specific training in conducting interviews through direct clinical work and formal research education in qualitative methods. This dual background supported the development of rapport and facilitated sensitive discussions around injury and emotional well-being. Throughout the research process, the interviewer engaged in reflexive practices, including peer and advisor debriefing, to acknowledge and mitigate potential biases and to enhance the trustworthiness and credibility of the data collection and interpretation.

### Procedure

Following approval from the Institutional Review Board (IRB), researchers obtained letters of support from participating schools. At pre-season football meetings, researchers introduced the study to the players and parents. Once parental consent was obtained, participants were enrolled in the study, and pre-season baseline data were collected, including a health history questionnaire for self-reported demographic information. Researchers contacted athletic trainers and research coordinators to identify athletes in the study who met the injury inclusion criteria. Identified participants were contacted, provided with a study information sheet, and invited to participate in the interviews. They were given a chance to ask questions and those who agreed scheduled a Zoom interview. Participants had the option to decline any questions and the researcher was available post-interview for questions and emotional support.

### Data analyses

Participant interviews were recorded, transcribed, and then analyzed using a thematic analysis approach (45), a qualitative descriptive method suitable for exploring multiple injury experiences due to its ability to provide a detailed examination of personal narratives. An inductive approach was employed, following Clarke and Braun's method (45), wherein initial descriptive coding identified basic, lower-order codes across interviews. Subsequent coding incorporated observational data, with higher-order themes developed to capture relationships between codes. To ensure internal homogeneity, researchers verified that the data within each theme were coherent and meaningfully related. This involved cross-checking the consistency of codes and themes within individual interviews and across different participants. External homogeneity was established by ensuring that the themes were distinct from each other and did not overlap significantly, thereby maintaining clear boundaries between different thematic categories. Findings were linked to research questions and relevant theoretical constructs, following established practices in sport psychology research [e.g., (46–48)]. Results are presented on common themes within and across participants for each interview construct and overall emergent themes.

### Transparency and Openness

Researchers went through the IRB protocol and were approved to conduct the study. Data is securely and confidentially stored in accordance with APA guidelines, and the data that support the findings of this study are available from authors upon reasonable request.

## Results

Eight high school football players who sustained multiple injuries during the season and were out of play for at least one week and/or one contest for each injury independently were interviewed. As well as describing their injuries and recovery, participants gave accounts of their experiences being injured and the impacts on their lives. After analyzing the data, four overarching themes emerged: (a) emotional response, (b) sources of support, (c) stress effects, and (d) coping strategies. Though these themes aligned well with the research questions, they were not predetermined by the interview structure but rather emerged organically from the data, representing an inductive approach. Each theme has subthemes, which are outlined in Table 2.

**Table 2:** Themes from multiply injured football players.

Theme	Subtheme	Description
Emotional response	(a) Grief (b) Aggravation (c) Minimizing	A reaction to injuries that elicits explicit feelings and patterns of expressing those feelings
Sources of support	(a) Family (b) Team	Types of assistance and encouragement provided by one's social network throughout their injuries
Stress effects	(a) Cognitive disruption (b) School difficulty	Impact of increased stress due to injuries
Coping strategies	(a) Mindset (b) Flocking	Methods used to manage the stress, adversity, and challenges of their injuries

### Emotional response

The first theme of emotional response is denoted as a reaction to injuries that elicits explicit feelings and patterns of expressing those feelings. There were solely negative emotions elicited by participants delineated into subthemes of grief and aggravation. A pattern of emotional expression, minimizing, is also identified as a subtheme.

#### Grief

The subtheme of grief encompasses sadness and loss in response to their injuries. Tyler expressed his reaction to his second injury that occurred during his senior night: “I wasn't crying because of the pain. I mean, I felt the pain a bunch, but I was just crying because I thought I was done with this injury”. Several of the participants situated their grief as related to the loss of the time and energy they had put into getting where they were in football before their injuries. An example of this was said by Danny:

I was heartbroken that I put a lot of hard work in it, and I didn't really get to finish it…it was sad, too, because it was just around the time in the season when I was starting to get it, and I was starting to figure it out and putting in a lot of work, like I stayed after practice, probably 30 min to do extra stuff, but it was just a really bad time.

#### Aggravation

Aggravation encompasses participant's feelings of annoyance, frustration, and anger in response to their injuries. When acknowledging these feelings, some individuals directed their emotions toward specific targets, whether internally or externally. Mike spoke of being “frustrated” with himself for his first injury because he felt like he “could have definitely avoided it”. Whereas Danny had “anger” directed towards the medical system discussing his diagnostic process and saying he “should not have been out as long as I was out”. The feeling of missing out and being unable to physically participate was connected with aggravation. Zach expressed this sentiment, stating, “Definitely frustration because I hate to miss football. I mean, I love the sport, so it sucks to miss. So, a lot of frustration.”.

#### Minimizing

In addition to the emotions participants acknowledged directly, some engaged in minimizing or avoidance of their emotions. This was evident in various ways during the interviews. Some participants when asked about their emotions immediately shifted the focus to their physical reactions to their injuries, as exemplified by Hunter who stated:

I guess the concussion, I don't know, I was just kind of out of it. Immediately after, my head hurt. And my broke wrist I was in pain, and I first thought about how I probably won't be able to play much. That's about it.

Many of them used language to minimize their feelings such as prefacing emotional labels with “a little” and/or after discussing adverse emotions they would end their statement by turning it into something positive. For example, Andrew said: “I'd say I'm a little bit frustrated that I keep getting hurt and a little sad that I keep getting hurt, but then also happy that I'm still able to support my team with the injuries. I might not be able to play, but I can also go out and support my team.”. Then, the most extreme version of minimizing was seen by denying any effects of the injuries such as when Kyle said:

They've not really affected my life very much. I mean, they've put me behind in school a little bit, but I've got mostly caught up…I mean, they've all affected me in a certain kind of way, but other than that, I feel like I'm good.

### Sources of support

The second theme identified was sources of support and is defined as types of assistance and encouragement provided by one's social network throughout their injuries. All participants emphasized the significance of support from others in their experiences and recovery from injuries. While many had diverse sources of support, these were categorized into primary subthemes of family and team.

#### Family

Social support by family members was the most discussed source of support. Participants noted their family providing verbal and emotional support, especially from their parents. For example, Andrew said:

I'd say my parents, because they kept telling me it's okay that you're hurt and you're just going to have to fight to get that spot back after…Pushing me and kind of reminding me why I fought so hard for those positions to begin with.

Several participants mentioned the motivational role of their parents during their injuries. John expressed this stating, “My mom helped a lot. She just kept me motivated in a way. Just tell me to keep going.”. Consistent familial support proved crucial for them, with participants noting that their parents “never left my side” and were “always telling me to keep going”.

#### Team

Given that football is a team sport, it's expected that teammates and coaches offered different degrees of support during injuries. Participants noted receiving emotional and verbal support from their teams, who also helped them stay involved despite their injuries. Some participants had coaches and teammates who provided hope for them. Hunter noted his coach saying, “you'll get back, you'll still have like a few games left” and John said his team would say they “can't wait to have you back”. Team members also played a key role in motivating participants throughout their injuries. Zach said, “I wanted nothing more than to play with them one last time and that's what kept me the most motivated”. Danny also spoke to this with the perspective of unity and shared responsibility within a team and not wanting to “let them down, because someone had to fill my gap”.

However, this subtheme was also seen as a source of non-support for three of the athletes. They expressed that their coaches and teammates had adverse reactions to their injuries including not understanding, blaming, and diminishing them. Tyler said that some of his teammates “don't really necessarily understand that this injury is a big part of my life”. Andrew was heavily influenced by his team's reaction to his injuries saying, “They almost blamed me for getting hurt, bringing up that I'm always hurt you don't deserve the spot, or you don't deserve the helmet or pads or something that because you're always hurt.”. Finally, Danny discussed his peers' reaction to his injuries saying they “thought I was soft because of it…Like not believing that you're actually hurt and that you're just like skipping out on practice.”.

### Stress effects

In response to their injuries, all but one athlete experienced increased stress. The third theme of stress effects denotes the impacts of increased stress due to injuries. The primary effects were seen through cognitive disruption and school difficulty.

#### Cognitive disruption

Participants' injuries induced stress that affected their thoughts. Three types of cognitive disruptions were identified: fear of reinjury, questioning, and overthinking. Specifically, three participants spoke of spending significant cognitive energy concerning reinjury. A quote by Tyler illustrates this well:

It's pretty stressful just dealing with it. You know, like when you're healthy and you're going into a game, you wonder, like, could I get hurt this game? Or you just sort of start to think of all the negative things that could happen. And I think that stresses you out a little bit…I just kept thinking about like just the bad things that could happen and it just started to put a toll on me.

Subsequently, some participants began to question if the possibility of another injury would be worth continuing to play. Andrew said that it “Kind of made me not want to play anymore because if I get hurt then it's just going to be my fault for getting hurt. So why would I want to play and risk it?”. Similarly, Tyler questioned the effectiveness of his physical rehabilitation and John questioned his abilities if he was “going to be good when I come back? Or was it just luck”.

The final cognitive disruption was classified as overthinking. John expressed “now I have to be extra cautious when I do anything at all. Just be careful on my shoulder. I just have to think a lot more about everything I do.”. Hunter also noted the toll that overthinking took on him and that he “had trouble sleeping sometimes because I just think about it.”.

#### School difficulty

Congruent developmentally, school was heavily impacted by their injuries. Four participants clearly expressed having difficulties in school due to the added stress of their injuries. They described physical obstacles, such as being unable to write due to their injuries, and expressed feelings of falling behind, particularly due to cognitive impairment resulting from concussions. Andrew discussed the relationship between stress and school:

They made me a little bit more stressed, especially school wise, because I was getting behind in school and I knew that I had to keep my grades up or else I wouldn't be able to play whenever I wasn't injured… I started pushing everything, pushing school away, procrastinating a lot, making everything harder on myself because I was stressed that I was going to do bad… and gave up on it instead of trying to do it.

### Coping strategies

Participants experienced a range of impacts on their well-being due to their injuries, as outlined in the previous theme. The fourth theme, coping strategies, encompasses methods utilized to handle the stress, adversity, and challenges arising from their injuries. The primary coping strategies employed by participants were mindset and flocking.

#### Mindset

Seven participants discussed adopting an adaptive mindset as a primary coping strategy. They used phrases like “good mindset”, “keeping a good head on my shoulders”, and “positive outlook/attitude” to describe their approach to coping with their injuries. Mike stated:

Trying to keep a positive attitude. I think that's really the most important thing, talking to other people helps. And thinking that just because you got injured, it doesn't mean that your sporting career is over or anything. Just look forward to being able to play after your injury is healed.

In this subtheme, several key codes emerged to describe the focus of participants' mindsets. The first is acceptance, viewed as the ultimate step in coping with grief, wherein athletes find acceptance with their injuries and their effects. This mindset was the most commonly adopted by the athletes and manifested at varying degrees, including acceptance of the recovery process, the injuries themselves, and the emotions elicited by them. For instance, Danny described a difference in his mindset between his injuries, stating, “I knew I was hurt, so I just accepted it, but I never accepted it with the first one”. Andrew acknowledged accepting that he is “a little bit injury prone” and using it as motivation to prevent further injuries. Mike and Tyler discussed accepting injuries as a part of football. Acceptance was also facilitated through finding meaning or purpose for their injuries, observed through spirituality and trust in future utility. Zach coped through prayer, while Tyler expressed faith in knowing “that God had a plan for me.”. Some participants framed their injuries in terms of future usefulness, knowing how to handle reinjury or viewing injuries as minor setbacks, serving as lessons in resilience. John found purpose by stating, “You just have to remember that everything happened for a reason and don't let it drag you down too much. Just keep your head up, think of the future.”.

Several participants coped by focusing on the controllable aspects of their situation, directing their efforts toward activities like lifting, reading, and doing things they could at practices. Some expressed that as they observed physical improvements, they used them as motivation. Additionally, participants employed different orientations for their goals: some prioritized the process over the outcome, setting small, achievable goals for success, as noted by Zach who said, “I tried to keep as positive as I could and set little goals at a time.. celebrating each and every success as if it's my biggest.”. Conversely, others focused on the outcome over the process for motivation, such as Andrew who stated, “Once I started getting closer to the end of the recovery process, it kind of made me want to push more to get that spot back because I was getting close to being able to get it again.”

Participants also adopted a mindset of trusting the process, particularly when offering advice to future injured athletes. The three participants who expressed this appeared to draw from their own experiences, advising to take time, not rush, focus on coming back stronger, and ease back into activities. John's quote exemplifies the significance of faith in trusting the process:

Don't let it get you down too much. You'll be able to come back stronger than you were. And you'd rather play it safe and wait and wait to play, than go back in and get injured and make it be even worse, then you'll be out even longer. Just take time, do all your rehab, and you'll be back soon.

All these mindsets that the athletes expressed as instrumental to coping with their injuries can be classified as adaptive. They allow athletes to find functionality in their injuries, accept them, and use them for psychological resilience in the future.

#### Flocking

The term “flocking” draws on principles from social and evolutionary psychology, describing the stress response of seeking out social support during times of distress (49). While social support emerged as a theme, it was also recognized as a coping response employed by participants. They spoke of talking to family, hanging out with friends, and surrounding themselves with people who were supportive throughout their recoveries. John said that he “stayed around people who were thinking good things, like “stay optimistic, don't let little things bother you”. Just having a good group of friends helps a lot.”. Participants not only engaged in flocking themselves but also encouraged other injured athletes to seek support. This advice included urging them to seek support from medical providers, as Tyler emphasized: “Don't wait. If you're hurt, just go see the trainer right away. Like, there's nothing embarrassing about that, you're just trying to get better as an athlete.”. It was evident that encouragement to seek support stemmed from personal experience, highlighting flocking as a potential preventative measure for future injuries.

## Discussion

The present study intended to empirically explore the experiences of high school football players who have sustained multiple injuries. The findings from the thematic analysis reveal themes of emotional response, sources of support, stress effects, and coping strategies, aligning with constructs of the Biopsychosocial Model of Sport-Injury Rehabilitation (6). They highlight the interconnectedness among various factors including injury history, sociodemographic variables such as gender, psychological elements like affect and cognition, and social aspects such as support networks and environment. These findings not only shed light on the challenges faced by high school football players, but also provide valuable implications for the development of supportive interventions that can be tailored to the needs of multiply injured athletes.

Regarding the emotional experiences of these athletes, the results demonstrated a spectrum of feelings, ranging from grief to aggravation. Grief responses reflect their investment in their football careers, underscoring the impact of injuries on their athletic identity. This is consistent with the existing literature attributing feelings of grief to the loss of athletic identity and the pre-injury self (50, 51). Also, fear of reinjury is one of the most prominent emotional consequences of injuries outlined in prior research, showing significant challenges for rehabilitation (52). Conversely, aggravation emerged as frustration and anger. While this response is well cited in the literature amongst other emotional responses (26, 28), there needs to be research exploring the specific role of anger as a response to injuries. This is especially critical to understand in male athletes, given that anger is widely accepted in response to disappointment, whereas sadness is often viewed as a sign of weakness, reflecting traditional masculine norms (53). These standards of masculinity, which discourage emotional expression, could explain why many participants tended to minimize their emotions yet readily cited forms of anger, a socially acceptable response for men (54). Considering the Gender Role Conflict (GRC) paradigm, exploring how male athletes negotiate these societal expectations in the context of injury-related emotions can provide deeper insights into coping mechanisms and targets for intervention.

Aligning with social constructs of the model, the results of this study highlighted the importance of different support networks in athletes' recovery journeys. Family emerged as a key source of emotional encouragement and motivation, with parents especially playing a central role in strengthening athletes' resilience. This frequent reliance on family support aligns with previous research, such as Petrie et al. (55), which suggests familial support is the most important in enhancing athletes' efforts against injury related stress. The importance of social relationships was a consistent thread throughout the other themes as well. It was observed that athletes' relationships were strained by the stress of their injuries, and they sought support as a coping resource. This further validates the existing literature on the necessity of social support for injured athletes (35, 36).

However, the study also revealed instances of unsupportive reactions from coaches and teammates. Several potential explanations for this can be drawn from prior research. One explanation is the concept of “unrecognized grief”, in which the loss experienced due to injury isn't acknowledged, understood, or socially supported, leading to feelings of judgment and isolation (66). Another explanation could be linked to masculine norms prevalent in football culture, which prioritize physical toughness (56). Within such cultures, succumbing to injury may be seen as a display of vulnerability, which could be deemed unacceptable resulting in further isolation. This underscores the significance of nurturing a supportive team culture and promoting empathy within the athletic community.

The analysis of stress experiences and coping mechanisms reveals the diverse impact injuries have on these athletes' lives. A primary observation was injury stress led to disruptions in thinking processes. This aligns with the findings of Short et al. (29), which showed that athletes with prior injuries tended to believe they were more likely to get injured again, felt more worried, and had less confidence in avoiding reinjury. Furthermore, Christakou et al. (57) illustrated how cognitive disruptions, measured by re-injury worry, confidence, and attention, can predict the likelihood of future injuries throughout a season, highlighting the substantial impact of psychological factors on injury risk assessment. Given that prior injuries can result in increased cognitive disruption and potentially pose a risk for re-injury, there is a need to address and understand the unique needs of athletes with multiple injuries. Additional research should be conducted to explore differences in prevalence and magnitude of cognitive disruptions between first-time injured athletes and those with multiple injuries and assess for re-injury risks.

Despite these challenges, participants employed diverse coping strategies to manage the effects of their injuries. The benefits of acceptance and reappraisal have been shown to be beneficial to rehabilitation adherence and in building resilience in the face of injury (58, 59). The participants' focus on staying positive and finding meaning in their injuries corresponds with the idea of mental toughness; a relentless persistence in the face of adversity (60). This notion is pervasive in sports culture and can explain the adaptive coping strategies seen in this study. Furthermore, the frequent use of adaptive coping strategies could result from insight gained from prior injuries, prompting the need for further exploration of potential differences in coping between athletes with single and multiple injuries. It is also important to explore the differences between types of multiple injuries, such as concussions vs. musculoskeletal injuries, and their severity. Concussions, for instance, may present unique psychological challenges and longer-term cognitive effects compared to musculoskeletal injuries, which might involve more immediate physical pain and functional limitations. Understanding these distinctions can help tailor rehabilitation approaches to better address the specific needs of athletes based on the type and severity of their multiple injuries.

### Limitations

This study has several limitations to consider. First, most participants came from the same school, potentially limiting the diversity of team environments experienced, which might have affected the social support results. Additionally, the participant sample was relatively racially homogenous, indicating a need for more diverse samples to understand a broader range of experiences. Moreover, the varying timeframes of the post-injury interviews could have impacted the accuracy of accounts, as athletes further from their injuries may have had a more adaptive outlook compared to those newly injured. It's also important to note the interviews were conducted by an unfamiliar female researcher, which might have influenced the athletes’ willingness to express their authentic feelings. In relation to the biopsychosocial model, while the study addressed stress, it didn't explicitly explore its biological aspects, which could offer a more comprehensive understanding of the effects of multiple injuries. Future research could use a mixed-methods approach, combining assessments of stress biomarkers with psychological and social evaluations. An alternative biopsychosocial model that may align more closely with the study's findings is the framework proposed by Wiese-Bjornstal (61), which emphasizes the influence of psychological and sociocultural factors on injury risk, response, and recovery. Further research is warranted to explore the applicability of this model within the context of multiple injuries. Additionally, the study didn't investigate rehabilitation outcomes or the intermediate biopsychological effects of injuries, which could provide valuable insights into long-term outcomes. Despite these limitations, this study is an important first step in addressing a gap in the literature regarding the experiences of athletes with multiple injuries. It emphasizes the need for further comprehensive investigation into understanding and addressing the distinct needs of this population.

### Implications for practice

The present study's findings yield significant implications for practitioners supporting athletes with multiple injuries. Foremost, ensuring robust social support networks emerges as a critical factor in facilitating recovery by fostering interconnectedness among athletes and their support systems, along with promoting a culture of ongoing help-seeking behavior. This is particularly important for athletes with multiple injuries, as they face compounded physical and psychological challenges, such as prolonged recovery times, increased risk of re-injury, and heightened emotional stress. A robust support network is crucial to navigate these challenges. Some strategies to foster support and inclusion could involve developing structured plans that keep injured athletes actively engaged in team life—for example, assigning meaningful roles during practices (e.g., assisting with drills, tracking stats) or designating specific responsibilities on game days (e.g., sideline support, mentoring younger players).

Additionally, acknowledging the diverse emotional responses unique to multiple injuries (e.g., grief, aggravation, minimizing, etc.) underscores the importance of promoting emotional expression and fostering acceptance. These goals can be effectively addressed through therapeutic approaches like Emotion-Focused Therapy (EFT) or Acceptance and Commitment Therapy (ACT), which are designed to help individuals process complex emotions and develop adaptive coping strategies. In addition, incorporating mental skills training—such as visualization and imagery—can enhance adaptive coping by helping athletes maintain a sense of involvement and connection to their sport, even when physically sidelined (62, 63). Furthermore, targeting injury-induced cognitive disruptions consistent with multiple injuries —such as fear of reinjury, persistent questioning, and overthinking— can aid in further injury prevention efforts. These disruptions, which often manifest as intrusive thoughts, self-doubt, and heightened vigilance, can be targeted through interventions such as cognitive restructuring to challenge maladaptive beliefs, mindfulness-based practices to reduce rumination and anxiety, and guided imagery or exposure-based techniques to gradually reduce fear of reinjury. Incorporating these strategies into rehabilitation programs can help athletes regain cognitive clarity, rebuild confidence, and make more adaptive return-to-play decisions.

In sports like football, where injuries are common and a masculine environment prevails, it is vital to cultivate a culture that acknowledges the vulnerability associated with injuries and actively includes injured athletes to enhance team cohesion and support. Promoting this atmosphere within teams can be supported through interventions such as workshops on emotional literacy that reframe emotional expression and vulnerabilities as strengths. Additionally, routine check-ins within teams that normalize open dialogue about physical and mental challenges can help establish norms that recognize injuries as part of the game, reducing the sense of isolation for athletes who experience multiple injuries.

Importantly, male athletes may be particularly vulnerable to Gender Role Conflict (GRC), which can discourage emotional expression and help-seeking due to internalized norms around toughness and stoicism (54). This conflict can significantly hinder psychological recovery following injury. To address this, interventions grounded in Relational-Cultural Theory or Relationally Engaged (RE) practices (64)—which emphasize connection, empathy, and mutual support—can be integrated into team environments. Football, with its strong emphasis on brotherhood and loyalty, presents a unique opportunity to redefine masculinity in ways that support emotional expression. By leveraging the “brotherhood” dynamic—caring for injured teammates as “fallen soldiers” and supporting them through rehabilitation—teams can create a space where vulnerability is not only accepted but honored. Coaches and team leaders can play a pivotal role in modeling this shift by validating emotional experiences and encouraging peer support during recovery.

Moreover, leveraging an athlete's resilience cultivated through sport participation and experience with prior injuries can facilitate adaptive coping during injury rehabilitation. Specifically, for athletes with multiple injuries, it is crucial to understand the compounded effects these injuries can have on their physical and mental health. This understanding highlights the importance of an interdisciplinary team approach, involving clinicians, coaches, athletic trainers, and mental health professionals, to provide comprehensive care. Such a team can collaboratively address the multifaceted needs of these athletes, ensuring that all aspects of their well-being are considered. Ultimately, individualized care that addresses the unique needs of each injured athlete and considers the potential impacts of previous injuries on their recovery emerges as a cornerstone of effective rehabilitation practices. By integrating insights from various disciplines, practitioners can develop more holistic and effective strategies to support athletes through their recovery journeys.

The current study explored the complex experiences of high school football players with multiple injuries from a psychosocial perspective. It uncovered the profound impact of injuries on athletes’ identities and well-being, emphasizing the crucial role of social support networks. Challenges in injury acceptance within football teams highlight the need for more inclusive team cultures. Additionally, the analysis revealed significant stress among injured athletes, with cognitive disruptions potentially increasing the risk of reinjury. Despite these challenges, athletes demonstrated adaptive coping mechanisms. Overall, this study enhances our understanding of the psychosocial aspects of multiple injury experiences and calls for future research to develop targeted interventions to meet the unique needs of injured athletes.

## Data Availability

The raw data supporting the conclusions of this article will be made available by the authors, without undue reservation.
